# Physical Activity, Screen Time, and Dietary Intake in Families: A Cluster-Analysis With Mother-Father-Child Triads

**DOI:** 10.3389/fpubh.2018.00276

**Published:** 2018-09-28

**Authors:** Christina Y. N. Niermann, Sarah Spengler, Jessica S. Gubbels

**Affiliations:** ^1^Department of Sport Science, University of Konstanz, Konstanz, Germany; ^2^Department of Sport and Health Science, Technical University of Munich, Munich, Germany; ^3^Department of Health Promotion, Maastricht University, Maastricht, Netherlands

**Keywords:** health behavior, physical activity, dietary intake, screen time, family, cluster analysis, co-occurrence

## Abstract

**Background:** The co-occurrence of multiple health behaviors such as physical activity, diet, and sedentary behavior affects individuals' health. Co-occurence of different health behaviors has been shown in a large number of studies. This study extended this perspective by addressing the co-occurrence of multiple health behaviors in multiple persons. The objective was to examine familial health behavioral patterns by (1) identifying clusters of families with similar behavior patterns and (2) characterizing the clusters by analyzing their correlates.

**Methods:** Cross-sectional data were collected from 198 families (mother, father, and child). Mothers, fathers, and children completed questionnaires assessing health related behaviors (physical activity, consumption of “healthy” and “unhealthy” foods, and screen time), the perception of Family Health Climate (regarding physical activity and nutrition) and demographics. Twelve variables (four health behaviors of three family members) were included in a cluster analysis conducted with Ward's Method and K-means analysis. Chi-square tests and analyses of variance were performed to characterize the family clusters regarding their demographics and their perception of Family Health Climate.

**Results:** Three clusters of families with specific behavioral patterns were identified: “healthy behavior families” with levels of physical activity and consumption of healthful foods above average and levels of media use and consumption of sweets below average; “unhealthy behavior families” with low levels of consumption of healthful foods and high levels of screen time; “divergent behavior families” with unhealthier behavioral patterns in parents and healthier screen time and eating behaviors combined with low physical activity levels in children. Family Health Climate differed between family clusters with most positive ratings in “healthy behavior families” and least positive ratings in “unhealthy behavior families.” “Divergent behavior families” rated the nutrition climate nearly as high as “healthy behavior families” while they rated the physical activity climate nearly as low as the “unhealthy behavior families.”

**Conclusions:** The study shows that co-occurrence of multiple health behaviors occurs on the family level. Therefore, focusing the family as a whole instead of individuals and targeting aspects related to the Family Health Climate in interventions could result in benefits for both children and adults and enhance effectivity of intervention programs.

## Introduction

Several health-related behaviors such as physical activity, diet, and sedentary behavior, contribute to health and health restrictions, respectively (1). Mostly, it is not one of these behaviors but the combination of multiple behaviors that affects individuals' health. Synergistic effects of different lifestyle behaviors might occur that affect the development of different health outcomes (2).

### Co-occurrence of health behaviors

A large number of studies examined the co-occurrence of different health behaviors within individuals (3). Clustering of health related behaviors has been shown in children [e.g., (4, 5)], adolescents [e.g., (6–10)], and adults [e.g., (3, 11)]. Different combinations of behaviors have been studied, ranging from more narrow patterns, such as patterns of dietary intake or physical activity [e.g., (12–14)], to broader lifestyle patterns such as energy balance-related patterns (4, 10, 15) or health risk behavior patterns (2, 3, 7, 16). Multiple behavioral patterns have been associated with different health outcomes, for example metabolic disorders (e.g., type 2 diabetes) and cardiovascular risk factors (13), psychosocial problems (7), or self-rated health (17, 18). Furthermore, clustering of diet, physical activity, and sedentary behavior was associated in several studies to overweight and obesity [e.g., (6, 8, 17, 19)], but overall the results were inconsistent (9).

It is well-known that individuals' healthy lifestyles are determined by a multitude of influences, including individual as well as social and physical environmental factors and their interplay. Individuals' health behavior patterns develop and maintain not in a vacuum. Health behaviors are embedded in social contexts and are affected by social ties (20, 21). One of the most important social contexts is the family.

### Health and health behavior within families

Behavior-related risk factors tend to accumulate within families (22). Resemblances have been shown for example with regard to weight status (23, 24), cardiovascular risk factors (25, 26), and abdominal visceral fat (27). Accordingly, several studies have shown familial resemblance regarding the underlying behaviors. Regarding dietary intake, overall low to moderate parent-child resemblances were shown, with differences depending on children's age and gender, type of parent-child dyad as well as food groups (28–30). For instance, moderate correlations between parents and children have been found for consumption of fruit whereas lower correlations have been found for sweets (28, 31–36). Beydoun and Wang (28) found stronger correlations in mother-child dyads compared to father-child dyads whereas other studies showed relevant correlations for father-child dyads (35) which were stronger compared to mother-child correlations (37, 38). Whereas many studies focused the parent-child relationship (mostly mother-child) there are also several studies showing significant moderate to strong correlations between dietary intake of spouses [e.g., (30, 39, 40)].

Similarly, the results regarding familial resemblance of physical activity and sedentary behavior vary due to differences in measurement issues, types of physical activities and sedentary behaviors, types of dyads and children's age and gender. Some studies showed that familial resemblance tends to be higher for sedentary behavior than for moderate to vigorous physical activities (41–43) whereas others found higher correlations for sports participation than for leisure-time activities including sedentary behaviors (44). Craig et al. (45) and Jacobi et al. (46) found moderate parent-child associations in objectively measured overall physical activity whereas in the study of Jago et al. (43) objectively measured physical activity was not associated. Several studies found differences depending on the type of dyads: some showed correlations only in mother-child dyads (46, 47), other studies found correlations between father-son and mother-daughter and lower correlations for parent-child dyads of opposite genders (48, 49). However, Maia et al. (44) found equal correlations for all parent-child dyads. Findings regarding spouse and sibling correlations are inconsistent, too: some found no correlations between spouses and higher correlations in siblings (46) whereas others found higher spouse than parent-child correlation and lower sibling correlations (44). Gomes et al. (50) found higher intra-generational similarities (between spouses or between siblings) than inter-generational similarities (parent-child).

Overall, literature indicates that there is familial aggregation of dietary intake, physical activity, and sedentary behavior patterns. However, the studies and results are heterogeneous and it is not possible to draw a clear conclusion [e.g., (29, 51)].

Taking into account the co-occurrence of health related behaviors within individuals and the resemblance of those behaviors within families, it could be worth to expand this multiple behavior perspective to the family level. To the best of our knowledge there are hardly any studies taking into account multiple behaviors of different family members simultaneously. Cameron et al. (15) found similar health behavior clusters (physical activity, sedentary behavior, positive and negative dietary behavior) in children and their mothers. They concluded that the concordance of these clusters indicates the importance of modeling and creating a healthy environment. However, clusters analyses were conducted separately for children and mothers. Davison and Birch (52) identified obesogenic and non-obesogenic families based on mothers' and fathers' physical activity and dietary patterns, children's behavioral patterns were not included. Daughters from families in the obesogenic cluster were at higher risk of overweight, showing greater increases in body mass index and skinfold thickness between ages five and seven. The greater increases in body mass index maintained across ages seven to 11 (53). As yet, there are no previous studies looking at clustering between multiple family members.

### Objective

Assuming that family members' behaviors are reciprocally related (54), this study aims to examine familial health behavioral patterns based on family member triads, namely mother, father, and child. The purpose of the study was (1) to identify clusters of families with similar behavior patterns including physical activity, screen time (as one subdomain of sedentary behavior that has been shown to be associated with different health indicators in adolescents and adults such as body composition, fitness, cardiovascular disease, and metabolic syndrome (55–57) and consumption of “healthy” and “unhealthy” foods of mother, father, and adolescent of each family and (2) to characterize the clusters by analyzing their correlates.

## Methods

The current study, named “Family and Health-Study,” was conducted in April 2012. The study was part of the multidisciplinary project EATMOTIVE funded by the German Federal Ministry of Education and Research.

### Procedure and participants

Participants were recruited via 11 secondary schools in the district of Konstanz, Germany. Students from the seventh grade upwards (corresponding to an age of at least 12 years) were approached. After making an appointment with the schools' principal, classes chosen by the principal were visited. The students were informed about the aims and requirements of the “Family and Health-Study” and received an envelope with three questionnaires: one for themselves, one for their mothers, and one for their fathers. In total, 1,500 students were approached. The students were asked to forward the questionnaires to their parents and were informed that each person needed to complete the questionnaire individually. Within 1 week, the students and their parents returned the completed questionnaires in sealed envelopes to their class teacher and the envelopes were collected by a member of the research team. In 317 families, at least one family member filled in the questionnaire (315 children, 288 mothers, 225 fathers). In 198 families, all three family members (mother, father, and child) living in the same household completed the questionnaires. Due to missing data on the individual level nine families were further excluded and finally 189 mother-father-child triads were included in the cluster analyses.

### Ethics statement

A full review and an approval of this study by an ethics committee was not required according to local and national guidelines. This research is exempt from institutional review board review according to the German Research Foundation [Deutsche Forschungsgemeinschaft (DFG)]^1^ and the National Science Foundation [National Science Foundation (NSF)]^2^ The study included an anonymous survey that did not involve collection of identifiable data. The survey was purely observational (non-invasive, non-interactive) and did not induce any type of psychological stress or anxiety. The participants were not member of a vulnerable group.

The study protocol was defined by a multidisciplinary expert panel of scientists involved in the EATMOTIVE project. The study fully conformed to the Declaration of Helsinki and the ethics guidelines of the German Psychological Society. The researchers visited the classes and briefly introduced the study to the students and the teachers. The students received detailed information for themselves and their parents regarding voluntary participation, handling of the questionnaires and processing of their data according to the ethics guidelines of the German Psychological Society (Deutsche Gesellschaft für Psychologie)^3^ Written informed consents were obtained from the parents of the participating students. The written consents were returned to the teachers. Apart from this, there was no interaction between the researchers and the participating students or parents. The participants had the opportunity to opt out of the study until the sealed envelopes were returned to the teachers. Thereafter, it was no longer possible to assign the questionnaires to a family.

### Measures

#### Clustering variables

##### Physical activity

Adolescents, mothers and fathers completed two items to assess their habitual physical activity (58). Corresponding to the guideline of the World Health Organization (59) the number of days with at least 60 min for the adolescents and 30 min for the mothers and fathers of moderate-to-vigorous physical activity during a “normal week” and during the “last week” were captured (“Over the past 7 days, on how many days were you physically active for a total of at least 60 (30) min per day?” and “Over a typical or usual week, on how many days are you physically active for a total of at least 60 (30) min per day?”). Responses were scored on an 8-point scale ranging from 0 to 7 days. According to the recommendations the mean of both items was calculated (58).

##### Screen time

Adolescents, mothers and fathers were asked how much time they spend watching TV and on the computer or internet on a typical day. Participants separately estimated the minutes for weekdays and weekend days. Minutes per week spending on TV and computer/internet were calculated by multiplying the minutes per weekday with five and adding minutes per weekend day multiplied by two.

##### Dietary intake

To obtain information on the consumption of healthful foods, adolescents, mothers and fathers completed a Food Frequency Questionnaire (60). The participants answered the question “How often do you normally eat the following foods?” for 25 food items. Salad, vegetables and fruit represented “healthful foods” according to the recommendations “10 rules of healthful eating” specified by the German Society of Nutrition (61). Chocolate, cake, and candies represented the category “sweets.” The consumption of those food items was rated on a 7-point Likert-type scale (“never,” “approximately one time per month,” “several times a month,” “approximately one time a week,” “several times a week,” “every day,” “several times a day”). The mean was calculated of the ratings of salad, vegetable and fruit and of chocolate, cake, and candies representing the consumption of healthful foods and the consumption of sweets.

#### Characterizing variables

##### Demographics

Adolescents, mothers, and fathers completed questionnaires to obtain demographic information including age, gender, educational level, and family structure. Adolescents stated which school they attended and which persons live currently in the household (categories: “mother,” “father,” “siblings,” “grandmother,” “grandfather,” and “others”). Mothers and fathers stated their marital status which was categorized into “living alone” and “living in a partnership/marriage in the same household” and “living in a partnership/marriage not in the same household.”

Parents' educational level was assessed by asking for the highest school qualification. According to the German tripartite school system the categories ranged from “no qualification” to “university-entrance diploma.” Employment status was categorized in “full-time,” “part-time,” “on parental leave,” “homemakers,” “unemployed,” “retired,” and “other”.

##### Family health climate

The FHC is a family level variable reflecting an aspect of the shared family environment (62, 63). It is defined as the shared perceptions and cognitions concerning a healthy lifestyle within a family. Therefore, we assume differences in the perception of the FHC between the family clusters. The Family Health Climate was assessed with the FHC-Scales for physical activity (FHC-PA) and for nutrition (FHC-NU) using a validated questionnaire (62). The FHC-PA Scale consists of 14 items pertaining to three subscales (*value*, e.g., “In our family it is normal to be physically active in our leisure time”; *cohesion*, e.g., “…we have fun doing physical activities together (e.g., bike tours, hikes),” and *information*, e.g., “…we collect information (e.g., on the internet) on physical activity and exercise”). The FHC-NU Scale comprises 17 items pertaining to four subscales (*value*, e.g., “…it is normal to choose healthful foods,” *cohesion*, e.g., “…we appreciate spending time together during meals,” *communication*, e.g., “…we talk about which foods are healthful,” and *consensus*, e.g., “…we rarely argue about food- or diet-related matters”). The items were rated on a 4-point Likert-type scale ranging from 0 = “not true” to 3 = “true.” Scores representing the mean of all items were calculated for the FHC-PA and FHC-NU, respectively. Mothers, fathers and adolescents each completed the scales individually. The internal consistencies in this study were α_FHC−PA_ = 0.92 and α_FHC−NU_ = 0.86 for mothers, α_FHC−PA_ = 0.90 and α_FHC−NU_ = 0.86 for fathers, and α_FHC−PA_ = 0.90 and α_FHC−NU_ = 0.85 for adolescents. The overall FHC was calculated as the sum of the individual scores of child, mother and father (FHC_agg_). The FHC_agg_ scores ranged between 0 and 9 and reflect the climate score within the family across its members (64).

### Analyses

Statistical analyses were performed with IBM SPSS Statistics version 22 (IBM Corp., NY, USA). For all variables, <5% of values were missing. Missing data were imputed using the expectation maximization algorithm after checking that missing values were completely at random using Little's MCAR test (65). Item distributions were inspected for multivariate normality. Skewness and excess of all items were below the thresholds of 2 and 7, respectively, as suggested by Curran, West, and Finch (66).

Aiming at identifying groups of families with similar behavior patterns, cluster analyses were used (67). Cluster analysis groups a set of objects in such way that objects in the same cluster are more similar to each other in terms of the included variables than to those in other clusters. In our analyses mother-father-child triads were arranged as objects. Therefore, cluster analyses were performed on the family triad level resulting in clusters of similar family triads. Twelve variables were included in the analyses: activity level of mother (1), father (2), child (3), screen time (TV, PC) of mother (4), father (5), child (6), consumption of healthy food (fruit, vegetable, and salad) of mother (7), father (8), child (9), and consumption of sweets (chocolate, cake, and sweets) mother (10), father (11), child (12). Behavioral variables were standardized with z-scores. A combination of hierarchical and non-hierarchical cluster analyses was used, following the recommendations of Punj and Stewart (68). First, Ward's method was performed based on squared Euclidean distances. The decision on optimal number of clusters was empirically funded by visual inspection of the dendogram and investigation of the error sum of squares increase (Elbow method) (69). On this basis, content interpretation was taken into account for the final decision which is requested for cluster analyses (70). To optimize the classification, the chosen cluster solution was taken as a starting partition for K-means analysis, which further fine-tunes the classification. All analyses were repeated with a randomly selected subsample (50%) to test reliability and stability of the cluster solution. Homogeneity was assessed as the percentage of consistently allocated participants (in K-means analysis compared to Ward's method) and by comparing variance of an item within clusters with variance of the item within the entire sample.

ANOVAs and chi square tests were performed to determine differences between the clusters. Intraclass correlation coefficients (ICC, one-way random, absolute agreement) were calculated to examine the similarity between family members within families (mother-father-child triads).

## Results

The children had a mean age of 14.02 years (*SD* = 1.17 years), 61.9% were female. More than 70% (*n* = 139) of the adolescents attended the highest level of secondary school (“Gymnasium” in the German tripartite school system) the rest attended lower levels of secondary schools (“Realschule,” “Werkrealschule,” and “Hauptschule”). The mothers had a mean age of 45.15 years (*SD* = 4.24 years; range 34–56 years). Fifty-six (29.6%) mothers had a university-entrance diploma (“Abitur”) and 21 (11.1%) had an advanced technical college certificate (“Fachhochschulreife”). At the time of the study, 24 (12.7%) mothers worked full-time, 135 (71.4%) worked part-time, and the rest (15.4%) were unemployed, retired, on parental leave, homemakers or had another employment status. The fathers had a mean age of 47.81 (*SD* = 6.43 years, range 27–74 years). Seventy-two (38.1%) fathers had a university-entrance diploma and 37 (19.6%) had an advanced technical college certificate. At the time of the study, 170 (89.9%) fathers worked full-time, 5 (2.6%) worked part-time, and the rest (7.4%) were on parental leave, unemployed, retired, homemakers, or had another employment status.

### Clustering of family members' health behaviors

Three clusters of families with specific behavioral patterns were identified. The stability test showed moderate agreement (Kappa = 0.57). 84.7% of the families were consistently allocated to the same cluster across the two conducted analyses, which shows a good overall homogeneity. For clusters 1 and 3, the variance of the items within clusters was satisfactorily small for most items, which further shows good homogeneity in these clusters. In contrast, for cluster 2 the variance of the items within the cluster was higher than the variance of the respective items within the entire sample for 10 out of 12 items. This fact indicates a weaker homogeneity for cluster 2.

**Figure 1:**
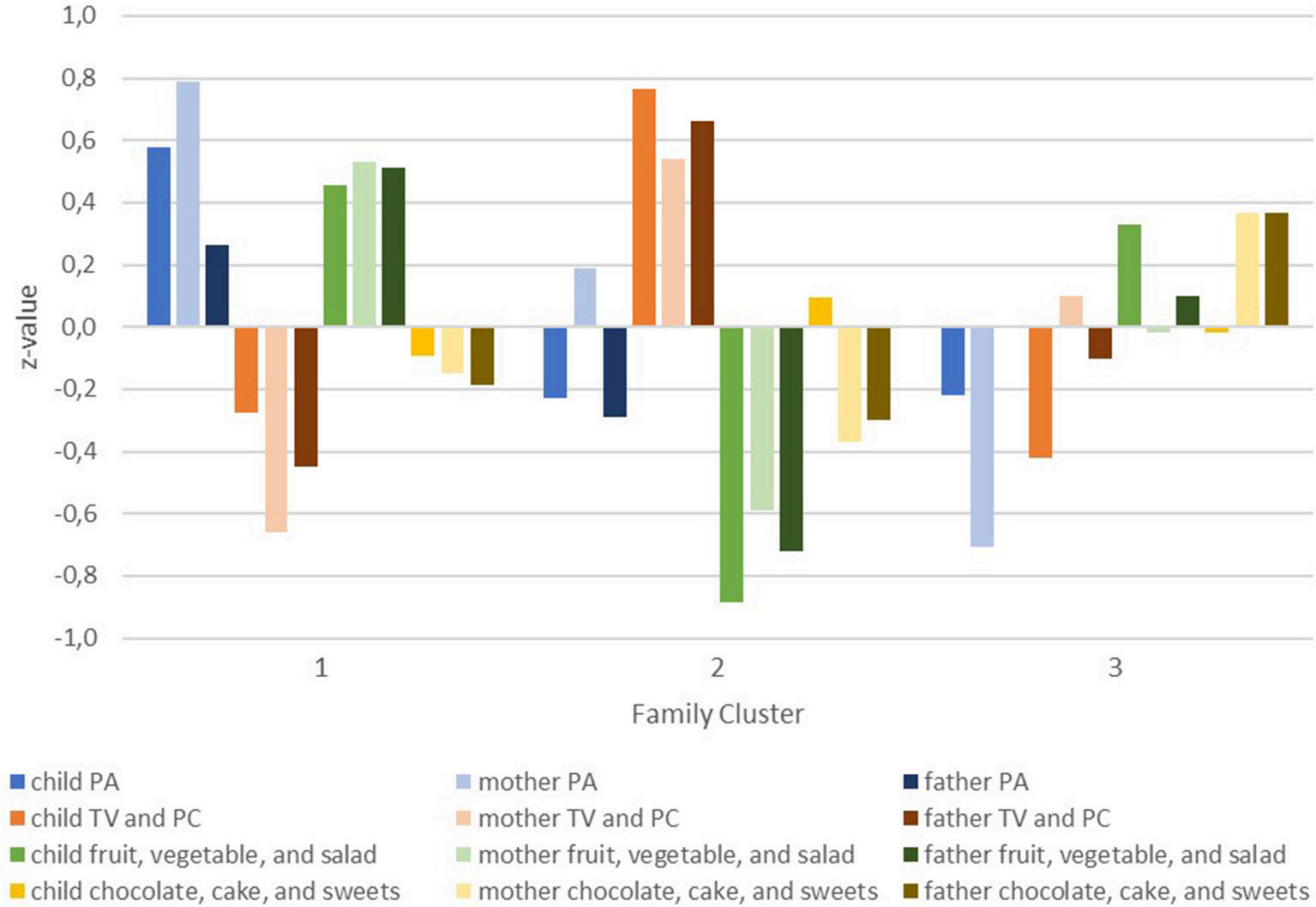
Behaviors (z-values) of children, mothers, and fathers per family cluster.

Figure 1 shows the behavioral patterns indicated by z-scores of children, mothers, and fathers within the three clusters.

In the first cluster of families, children, mothers, and fathers self-reported the healthiest behavioral patterns. The “healthy behavior families” cluster is characterized by physical activity levels and consumption of healthful foods above average (z-scores above 0) and media use and consumption of sweets for children, mothers, and fathers below average. The second cluster comprises families with unhealthy behavioral patterns and is therefore termed “unhealthy behavior families.” Especially low consumption of healthful foods (below average for all three family members) and high levels of screen time (above average for all three family members) are conspicuous in this cluster. Their physical activity and intake of sweets shows a less consistent pattern across family members. The third cluster is termed “divergent behavior families.” Children, mothers, and fathers differ in their health behavioral patterns. On average the mothers in this cluster have unhealthier patterns (levels below average for physical activity and healthful foods and above for screen time and sweets) while the children show healthier screen times and eating behaviors combined with low physical activity levels. Results for the four behavioral categories of children, mothers and fathers in the family clusters are presented in Table 1.

**Table 1:** Individual health behaviors within family clusters.

		**Cluster**			***F*-Test (*F, p*, eta^∧^2)**
		**1 (*n* = 58)**	**2 (*n* = 57)**	**3 (*n* = 74)**	
		***M* (*SD*)**	***M* (*SD*)**	***M* (*SD*)**	
Healthful foods (fruit + salad + vegetable)^*a*^	Child	13.53 (1.67)	10.11 (2.77)	13.21 (1.78)	47.92, < 0.001, 0.34^+^
	Mother	14.22 (1.26)	12.04 (2.15)	13.16 (1.76)	22.18, < 0.001, 0.19^+^
	Father	13.51 (1.54)	10.79 (2.45)	12.60 (1.74)	29.82, < 0.001, 0.24^+^
Sweets (chocolate + candy + cake)^a^	Child	9.73 (2.46)	10.30 (3.39)	9.95 (3.16)	0.51, 0.601
	Mother	8.64 (3.00)	7.96 (3.13)	10.21 (2.62)	10.52, < 0.001, 0.10^+^
	Father	8.48 (3.81)	8.10 (3.51)	10.39 (2.70)	9.15, < 0.001, 0.09^+^
PA^b^	Child	4.84 (1.40)	3.56 (1.58)	3.57 (1.40)	15.22, < 0.001, 0.14
	Mother	5.91 (1.14)	4.76 (1.88)	3.05 (1.35)	62.99, < 0.001, 0.40^+^
	Father	4.66 (1.87)	3.58 (2.04)	4.14 (1.92)	4.47, 0.013, 0.05
TV + PC (minutes per week)^c^	Child	14.23 (8.91)	26.96 (14.13)	12.45 (7.02)	36.65, < 0.001, 0.28^+^
	Mother	7.51 (5.06)	16.44 (7.48)	13.15 (6.73)	27.82, < 0.001, 0.23
	Father	10.58 (6.02)	19.28 (9.39)	13.31 (5.55)	22.98, < 0.001, 0.20^+^

a Rated on 7-point Likert-type scale: 0 = “never,” 1 = “approximately one time per month,” 2 = “several times a month,” 3 = “approximately one time a week,” 4 = “several times a week,” 5 = “every day,” 6 = “several times a day.”

b *Days per week with more than 30/60 min PA*.

c *Hours per week (7 days) with TV and PC*;

+ *no variance homogeneity*.

### Resemblances within families

Intraclass-correlations per cluster are presented in Table 2. In all three clusters highest resemblances were found for consumption of healthful foods. Lowest ICCs were found for physical activity levels and media use in “unhealthy behavior families” and in “divergent behavior families.” Overall, children, mothers, and fathers in the “healthy behavior families” cluster have more similar behaviors than in “unhealthy behavior families” and “divergent behavior families.”

**Table 2:** Resemblances of child's, mother's, and father's behaviors within the clusters.

	**Cluster 1**		**Cluster 2**		**Cluster 3**	
	**ICC (1,1)^a^**	**ICC (1,3)^b^**	**ICC (1,1)^a^**	**ICC (1,3)^b^**	**ICC (1,1)^a^**	**ICC (1,3)^b^**
Healthful foods	0.19^*^	0.42^*^	0.19^*^	0.41^*^	0.13^*^	0.30^*^
Sweets	0.23^*^	0.47^*^	0.15^*^	0.34^*^	0.03	0.08
PA	0.11	0.27	0.07	0.19	0	0
TV + PC	0.21^*^	0.44^*^	0	0	0.07	0.19

a *Single measure*.

b *Average measure*,

* *p < 0.05*.

### Characterization of family clusters

The association of the clusters with demographic variables and families' perception of Family Health Climate are presented in Table 3. Significant differences were found for educational levels of children and mothers but not fathers. The “healthy behavior families” and “divergent behavior families” are higher educated. Nearly 75% of the mothers in the “unhealthy behavior families” have the intermediate and lowest educational level. There was a significant difference regarding the number of children in the families: “healthy behavior families” have more children, while the “unhealthy behavior families” and the “divergent behavior families” have fewer children.

**Table 3:** Association of the clusters with demographic variables and Family Health Climate.

		**Cluster**			
		**1**	**2**	**3**	
Gender	Child	*m* = 19 (32.8%) *w* = 39 (67.2%)	*m* = 29 (50.9%) *w* = 28 (49.1%)	*m* = 24 (32.4%) *w* = 50 (67.6%)	χ^2^*=* 5.66, *p =* 0.059
Secondary school type	Highest level (Gymnasium)	46 (83.6%)	34 (59.6%)	59 (86.8%)	χ^2^ = 20.72, *p* < 0.001
	Intermediate level (Realschule)	1 (1.8%)	13 (22.8%)	4 (5.9%)	
	Lowest level (Hauptschule)	8 (14.5%)	10 (17.5%)	5 (7.5%)	
Age	Child	14.07 (1.15)	14.21 (1.25)	13.82 (1.10)	*F* = 1.86, *p =* 0.159
	Mother	46.18 (4.42)	44.91 (4.32)	44.54 (3.92)	*F* = 2.57, *p =* 0.079
	Father	47.70 (5.39)	48.82 (8.70)	47.11 (4.91)	*F* = 1.16, *p =* 0.315^+^
Educational level mother	Highest level (Hochschul-/Fachhochschulreife)	26 (44.8%)	15 (26.3%)	36 (48.6%)	χ^2^ *=* 14.02, *p =* 0.029
	Intermediate level (Realschulabschluss)	25 (43.1%)	27 (47.7%)	29 (39.2%)	
	Lowest level (Hauptschulabschluss)	4 (6.9%)	14 (24.6%)	7 (9.5%)	
	Other	3 (5.2%)	1 (1.5%)	2 (2.7%)	
Employment status mother	Full time	7 (12.1%)	9 (16.4%)	8 (11.1%)	χ^2^*=* 1.75, *p =* 0.781
	Part time	39 (70.9%)	40 (72.7%)	56 (77.8%)	
	Currently not working	9 (16.4%)	6 (10.9%)	8 (11.1%)	
Educational level father	Highest level (Hochschul-/Fachhochschulreife)	34 (58.6%)	30 (54.5%)	45 (61.6%)	χ^2^ *=* 7.78, *p =* 0.254
	Intermediate level (Realschulabschluss)	12 (20.7%)	12 (21.8%)	13 (17.8%)	
	Lowest level (Hauptschulabschluss)	8 (13.8%)	13 (23.6%)	14 (19.2%)	
	Other	4 (6.9%)	0	1 (1.4%)	
Employment status father	Full time	50 (89.3%)	50 (89.3%)	70 (94.6%)	χ^2^*=* 2.76, *p =* 0.598
	Part time	2 (3.6%)	1 (1.8%)	2 (2.7%)	
	Currently not working	4 (7.1%)	5 (8.9%)	2 (2.7%)	
Number of children in the family	One child	4 (7.2%)	14 (24.6%)	11 (15.1%)	χ^2^ *=* 15.72 *p =* 0.015
	Two children	32 (57.1%)	26 (45.6%)	45 (61.6%)	
	Three children	11 (19.6%)	15 (26.3%)	14 (19.2%)	
	Four and more children	9 (16.1%)	2 (3.5%)	3 (4.1%)	
Family FHC-PA^a^		5.36 (1.20)	4.62 (1.54)	4.85 (1.38)	*F* = 4.50, *p =* 0.012, eta^∧^2 = 0.05^+^
Family FHC-NU^b^		6.71 (0.93)	5.87 (0.89)	6.46 (0.88)	*F* = 13.45, *p* < 0.001, eta^∧^2 = 0.13

The FHC was rated on a 4-point Likert-type scale ranging from 0 = “not true” to 3 = “true.”

+ *No variance homogeneity*.

a *Mean FHC-PA child + mean FHC-PA mother + mean FHC-PA father*.

b *Mean FHC-NU child + mean FHC-NU mother + mean FHC-NU father*.

As assumed the FHC for nutrition and physical activity was perceived most positive in “healthy behavior families” and least positive in “unhealthy behavior families.” *post-hoc* analyses (FHC NU: Gabriel's procedure; FHC PA: Games Howell) revealed differences in the nutrition climate between “healthy behavior families” and “unhealthy behavior families” (*p* < 0.001) and between “divergent behavior families” and “unhealthy behavior families” (*p* < 0.01) but not between “healthy behavior families” and “divergent behavior families” (*p* = 0.24). The physical activity climate differed significantly between “healthy behavior families” and “unhealthy behavior families” (*p* = 0.01) (“healthy behavior families” and “divergent behavior families”: *p* = 0.06).

## Discussion

The first aim of this paper was to explore the existence of health behavioral patterns on the family level. Clustering of health behaviors has been shown in a large number of studies [e.g., (3, 9)], but there are hardly any studies focusing on family clusters by including health behaviors of two or more family members simultaneously (15, 52). We included health behaviors of three persons per family (child, mother, and father) and identified three clusters that differ in their familial behavioral patterns.

### Family clusters: “healthy behavior families,” “unhealthy behavior families,” and “divergent behavior families”

The first cluster represents a “healthy” cluster including families with healthy behavioral patterns (with healthy eating and activity behaviors and lower levels of sweets consumption and media use) of all three persons. The second cluster is an “unhealthy” cluster with unhealthier behavioral patterns of the individuals in the families. Especially low consumption of healthful food and high levels of screen time of all three persons are conspicuous, with lowest and highest values, respectively, for children. “Healthy” and “unhealthy” clusters with different combinations of behaviors and different labels are found in many studies on the individual level [for an overview (3, 9)] e.g., in preschoolers (5) and adolescents (71). Studies on clustering of health behaviors in adults mostly include smoking and alcohol beside dietary intake, sedentary behavior, and physical activity [e.g., (72, 73)] which makes it difficult to directly compare the results with our finding regarding adults. However, a systematic review found that 81% of the identified studies reported a “healthy” cluster (3). Graham et al. (74) examined clustering of fruit and vegetable consumption, physical activity, smoking, and alcohol intake of mothers and their partners in England and found “unhealthy” patterns for both mothers and their partner. In addition, they analyzed the concordance of behavioral patterns of mothers and their partners and found a high degree of inter-couple concordance in health behaviors. Furthermore, Davison and Birch (52) found clusters termed “obesogenic” and “non-obesogenic” on the parental level. The “non-obesogenic” cluster comprises the combination of low energy intake levels with high levels of physical activity levels of both mothers and fathers. In contrast, the obesogenic cluster comprises parents with high energy intake and low physical activity levels. We are the first to examine clustering on the family level by including mother's, father's, and child's behaviors simultaneously. The results indicate that previously found “healthy” and “unhealthy” clusters also exist on the family level.

It should be noted that the “unhealthy” cluster in this study is not homogenous: different unhealthy behavioral patterns emerge in families in this cluster and it could be useful to have a closer look at the families in this cluster. A healthy lifestyle does not seem not to be important in these “unhealthy behavior families.” The FHC for both physical activity and healthy eating is rated lowest. The FHC represents a family level variable reflecting shared perceptions and cognitions regrading physical activity and healthy eating (63). It serves as a framework for an individual's everyday health behavior. Accordingly, the FHC for both physical activity and healthy eating is rated highest in “healthy behavior families.” In “divergent behavior families” the healthy eating FHC is rated high while the physical activity FHC is rated low. In line with this, “divergent behavior families” consume more healthful foods than “unhealthy behavior families” but have similarly low physical activity levels. Physical activity does not seem to be an important topic for these families. In the “divergent behavior families” cluster family members differ in their health behavior patterns. Interestingly, children's behaviors seem to be more healthful than parents' behavior; especially the mothers have unhealthy behavioral patterns. Although there seems to be unhealthy modeling especially from the mothers, the children in this cluster indicated a healthy dietary intake and the lowest levels of screen time. Parents do not have healthy behavior patterns themselves but it could be assumed that it is important for them, that their children have healthy behavioral patterns. Therefore, parents in this cluster might differ regarding their parenting practices and exert higher levels of control or restriction of screen time and make healthful food more and sweets less available or accessible.

The children in this cluster indicated a healthy dietary intake and the low levels of screen time combined with low levels of physical activity. Other cross-sectional studies found similar clusters for adolescent girls and boys (6, 10). Furthermore, the largest proportion of adolescents in the study of Ottevaere et al. (71) was in this mixed behavioral pattern cluster. In children and adolescents the pattern of low levels of sedentary behavior with low levels of physical activity is common (9).

### Family clusters and educational levels

In line with a large number of studies showing differences of health behaviors depending on socioeconomic status [e.g., (72, 75–78)], our “unhealthy” cluster is lower educated while the “healthy” cluster is higher educated. Unexpectedly, the “divergent behavior families” cluster is highly educated. It seems that mothers' educational level is most important for a healthy family life, as the clusters differ regarding maternal education but not paternal education. This relevance of maternal education could be related to the traditional role of mothers as main actors in family life, who are often primary responsible for example for providing healthy food via cooking or grocery shopping, and spend more time in interactions with their children (79–81). Although mother's employment and fathers' involvement in family life increase, this is still common in western families. Interestingly, the mothers in the “divergent behavior families” are highly educated, nearly 50% have the highest educational level, but they reported unhealthy behavioral patterns while their children reported healthy behavioral patterns (except physical activity). This might indicate that maternal educational level is related to the perceived importance of children's healthy behavioral patterns and to related knowledge regarding health behavior which might affect the use of related parenting practices (e.g., praising children more often for the consumption of fruit and vegetables) (82).

### Resemblances within family clusters

Resemblances of health behaviors are lowest in “divergent behavior families” while the “healthy behavior families” have the highest resemblances. Dietary intake (healthful food and sweets) and media use is associated between mothers, fathers, and children in these families while physical activity is not. Interestingly, mothers, fathers and children's consumption of healthy foods was consistently associated in all three clusters. It can be assumed that eating fruit, vegetables, and salad is more a family matter than for example being physically active, which might be done to a bigger extent outside family life. Additional analyses on dyadic resemblances (dyadic ICCs: mother-child, father-child, mother-father) revealed no clear pattern in the dyadic ICCs in the “healthy behavior families” and “unhealthy behavior families.” The ICCs differed depending on the behavior and the clusters. Similarities between mother-child was not consistently higher than for father-child as it was found in previous studies [e.g., (28)]. In contrast, in “divergent behavior families” the ICCs were higher for mother-father dyads than for both parent-child dyads. Parents' behaviors were thus more similar in these families than parent-child behaviors.

Co-occurrence of different health behaviors is complex (3, 9) and it is even more complex on the family level. Nonetheless, as our results demonstrated, it is worth to take into account co-occurrence of health behaviors on the family level. The cluster analysis clustered groups of families with more or less homogenous familial behavioral patterns. Although cluster two is not homogenous, it includes families with unhealthy behavioral patterns even though these unhealthy familial patterns differ between families. It could be assumed that, although the behaviors of the family members are less similar in “unhealthy behavior families” and “divergent behavior families,” there are underlying mechanisms and interaction patterns within families that influence individuals' behaviors (e.g., control, autonomy support by parents). Health behaviors are anchored in the family context: individuals' health behaviors are developed and maintained in daily family life (21, 54). In order to take into account the importance of the family, effective interventions targeting the promotion of children's health behaviors integrate specific modules aiming to educate parents and influence their parenting practices and styles (83–85). However, previous research has also shown that children influence parenting practices as well as the other way around (86). Furthermore, family-level socialization dynamics may affect the development and maintenance of a healthy or unhealthy lifestyle beyond the impact of dyadic interaction patterns between parents and children. Following the “families as systems” approach the family is more than the sum of the individuals and individuals' behavior cannot be understood in isolation from the rest of the system (87). Recent studies have recommended considering the overarching family context (63, 84, 88–90). Targeting the family as a whole instead of individuals in interventions could result in benefits for both children and adults.

### Strength and limitations

Even though this study was based on cross-sectional data a major strength of our study is the availability of data of 189 triads of both parents and their child.

We assessed physical activity and dietary behavior using short self-assessment questionnaires. It remains unknown if data based on objective measurements such as accelerometers would have resulted in different associations. The study suffered from a relatively low response rate (20%), which may have biased the results. Moreover, the sample was higher educated than the average German population possibly limiting the generalizability of the findings. Therefore, replicating the results in other samples or cultures is desirable.

In addition, generalization of findings to other populations is limited due to the explorative method of cluster analysis. Cluster analysis is a subject-centered and data-driven approach and results are in part based on the investigator's decisions. Hence, further studies are needed to augment evidence on the existence of the identified clusters.

Finally, we informed students and parents that the questionnaires should be completed individually. Although we carefully checked the plausibility of the data within families in the data clearing process, we were not able to control this aspect.

## Conclusion

Co-occurrence of different health-related behaviors has been shown in a large number of studies (91). This study extended this perspective by addressing the co-occurrence of multiple health behaviors in multiple persons: we showed the clustering of health related behaviors on the family level. Further studies should focus on underlying mechanisms that could explain why the behaviors of family members are interrelated (e.g., communication patterns, modeling, shared values etc.) which would be important for the development of family-based interventions. Despite the promising approach of addressing multiple health behaviors in interventions, it should be noted that studies evaluating multiple behavior interventions showed small effects (92). There is still the need to gain further knowledge on how behaviors are related or can be influenced simultaneously within interventions (93). This is in particular the case regarding the co-occurrence (and co-variation) of multiple health behaviors in different persons within families. As different unhealthy behavioral patterns exist within families that might be anchored in daily family life, the development of interventions focusing on the family as a whole and targeting aspects related to the Family Health Climate could enhance the effectivity of health promotion programs.

## Author contributions

CN was responsible for the overall conception, design and analysis of this study, and wrote the manuscript. SS conducted the statistical analyses, contributed to the interpretation of the study results and revised the manuscript. JG and SS contributed to the conception of the manuscript, to writing the manuscript, and they revised the manuscript. All authors were involved in critically revising the manuscript, and have given their approval for submitting the manuscript. All authors read and approved the final manuscript.

### Conflict of interest statement

The authors declare that the research was conducted in the absence of any commercial or financial relationships that could be construed as a potential conflict of interest.

## Footnotes

^1^Deutsche Forschungsgemeinschaft (DFG). Informationen für Geistes - und Sozialwissenschaftler/innen. (Accessed March 09, 2015). http://www.dfg.de/foerderung/faq/geistes_sozialwissenschaften/index.html

^2^National Science Foundation (NSF). Interpreting the common rule for the protection of human subjects for behavioral and social science research. (Accessed March 09, 2015). http://www.nsf.gov/bfa/dias/policy/hsfaqs.jsp#exempt.

^3^Deutsche Gesellschaft für Psychologie. Ethische Richtlinien der Deutschen Gesellschaft für Psychologie e.V. und des Berufsverbandes Deutscher Psychologinnen und Psychologen e.V. https://www.dgps.de/index.php?id=85.

## References

[1] Fisher EB Fitzgibbon ML Glasgow RE Haire-Joshu D Hayman LL Kaplan RM. Behavior matters. Am J Prev Med. (2011) 40:e15–30. 10.1016/j.amepre.2010.12.031 21496745 PMC3137947

[2] Berrigan D Dodd K Troiano RP Krebs-Smith SM Barbash RB. Patterns of health behavior in U.S. adults. Prevent Med. (2003) 36:615–23. 10.1016/S0091-7435(02)00067-1 12689807

[3] Noble N Paul C Turon H Oldmeadow C. Which modifiable health risk behaviours are related? A systematic review of the clustering of Smoking, Nutrition, Alcohol and Physical activity (‘SNAP’) health risk factors. Prevent Med. (2015) 81:16–41. 10.1016/j.ypmed.2015.07.003 26190368

[4] Gubbels JS van Assema P Kremers SPJ. Physical activity, sedentary behavior, and dietary patterns among children. Curr Nutr Rep. (2013) 2:105–12. 10.1007/s13668-013-0042-6 23638341 PMC3637646

[5] Miguel-Berges ML Zachari K Santaliestra-Pasias AM Mouratidou T Androutsos O Iotova V. Clustering of energy balance-related behaviours and parental education in European preschool children: the ToyBox study. Br J Nutrit. (2017) 118:1089–96. 10.1017/S0007114517003129 29198192

[6] Moreira NF da Veiga GV Santaliestra-Pasías AM Androutsos O Cuenca-García M Oliveira ASD. Clustering of multiple energy balance related behaviors is associated with body fat composition indicators in adolescents. Results from the HELENA and ELANA studies. Appetite (2018) 120:505–13. 10.1016/j.appet.2017.10.008 29017906

[7] Busch V van Stel H Schrijvers A de Leeuw J. Clustering of health-related behaviors, health outcomes and demographics in Dutch adolescents: a cross-sectional study. BMC Public Health (2013) 13:1118. 10.1186/1471-2458-13-1118 24305509 PMC3890495

[8] Carson V Faulkner G Sabiston CM Tremblay MS Leatherdale ST. Patterns of movement behaviors and their association with overweight and obesity in youth. Int J Public Health (2015) 60:551–9. 10.1007/s00038-015-0685-8 25985847

[9] Leech R McNaughton S Timperio A. The clustering of diet, physical activity and sedentary behavior in children and adolescents: a review. Int J Behav Nutr Phys Act. (2014) 11:4. 10.1186/1479-5868-11-4 24450617 PMC3904164

[10] Spengler S Mess F Mewes N Mensink GBM Woll A. A cluster-analytic approach towards multidimensional health-related behaviors in adolescents. BMC Public Health (2012) 12:1128. 10.1186/1471-2458-12-1128 23273134 PMC3552670

[11] Schneider S Huy C Schuessler M Diehl K Schwarz S. Optimising lifestyle interventions. Identification of health behaviour patterns by cluster analysis in a German 50+ survey. Eur. J. Public Health (2009) 19:271–7. 10.1093/eurpub/ckn144 19164433

[12] Jago R Fox KR Page AS Brockman R Thompson JL. Physical activity and sedentary behaviour typologies of 10-11 year olds. Int J Behav Nutr Phys Act. (2010) 7:59. 10.1186/1479-5868-7-59 20663226 PMC2918527

[13] Newby PK Tucker KL Empirically derived eating patterns using factor or cluster analysis: a review. Nutr Rev. (2004) 62:177–203. 10.1111/j.1753-4887.2004.tb00040.x 15212319

[14] Lee PH Yu YY McDowell I Leung GM Lam TH. A cluster analysis of patterns of objectively measured physical activity in Hong Kong. Public Health Nutr. (2013) 16:1436–44. 10.1017/S1368980012003631 22894896 PMC10271452

[15] Cameron AJ Crawford DA Salmon J Campbell K McNaughton SA Mishra GD. Clustering of obesity-related risk behaviors in children and their mothers. Ann Epidemiol. (2011) 21:95–102. 10.1016/j.annepidem.2010.11.001 21184950

[16] van Nieuwenhuijzen M Junger M Velderman MK Wiefferink KH Paulussen TW Hox J. Clustering of health-compromising behavior and delinquency in adolescents and adults in the Dutch population. Prevent Med. (2009) 48:572–8. 10.1016/j.ypmed.2009.04.008 19389423

[17] Spengler S Mess F Schmocker E Woll A. Longitudinal associations of health-related behavior patterns in adolescence with change of weight status and self-rated health over a period of 6 years: results of the MoMo longitudinal study. BMC Pediatr. (2014) 14:242. 10.1186/1471-2431-14-242 25270112 PMC4190442

[18] Conry MC Morgan K Curry P McGee H Harrington J Ward M. The clustering of health behaviours in Ireland and their relationship with mental health, self-rated health and quality of life. BMC Public Health (2011) 11:692. 10.1186/1471-2458-11-692 21896196 PMC3187756

[19] Gubbels JS Kremers SPJ Goldbohm RA Stafleu A Thijs C. Energy balance-related behavioural patterns in 5-year-old children and the longitudinal association with weight status development in early childhood. Public Health Nutr. (2012) 15:1402–10. 10.1017/S1368980011003089 22124196

[20] Umberson D Crosnoe R Reczek C. Social relationships and health behavior across life course. Annu Rev Sociol. (2010) 36:139–57. 10.1146/annurev-soc-070308-120011 21921974 PMC3171805

[21] Sallis JF Nader PR Family determinants of health behaviors. In: Gochman DS, editors. Health Behavior: Emerging Research Perspectives (New York, NY: Plenum Press) (1988). p. 107–24.

[22] Campbell TL Familien und Gesundheit: Zum Stand der Forschung. In: Kröger F Hendrischke A McDaniel S, editors. Familie, System und Gesundheit. Systemische Konzepte für ein soziales Gesundheitswesen (Heidelberg: Auer) (2000). p. 225–41.

[23] Hunt MS Katzmarzyk PT Pérusse L Rice T Rao DC Bouchard C. Familial resemblance of 7-year changes in body mass and adiposity. Obes Res. (2002) 10:507–17. 10.1038/oby.2002.69 12055327

[24] Maes HHM Neale MC Eaves LJ. Genetic and environmental factors in relative body weight and human adiposity. Behav Genet. (1997) 27:325. 10.1023/A:1025635913927 9519560

[25] Harrap SB Stebbing M Hopper JL Hoang HN Giles GG. Familial patterns of covariation for cardiovascular risk factors in adults: the victorian family heart study. Am J Epidemiol. (2000) 152:704–15. 10.1093/aje/152.8.704 11052548

[26] Brenn T. Adult family members and their resemblance of coronary heart disease risk factors: the cardiovascular disease study in finnmark. Eur J Epidemiol. (1997) 13:623–30. 9324207 10.1023/a:1007333919898

[27] Rice T Després JP Daw EW Gagnon J Borecki IB Pérusse L. Familial resemblance for abdominal visceral fat: the HERITAGE family study. Int J Obes Relat Metab Disord. (1997) 21:1024–31. 9368826 10.1038/sj.ijo.0800511

[28] Beydoun MA Wang Y. Parent–child dietary intake resemblance in the United States: evidence from a large representative survey. Soc Sci Med. (2009) 68:2137–44. 10.1016/j.socscimed.2009.03.029 19375837 PMC2730650

[29] Wang Y Beydoun MA Li J Liu Y Moreno LA. Do children and their parents eat a similar diet? Resemblance in child and parental dietary intake: systematic review and meta-analysis. J Epidemiol Commun Health (2011) 65:177–89. 10.1136/jech.2009.095901 21051779 PMC3010265

[30] Robinson LN Rollo ME Watson J Burrows TL Collins CE. Relationships between dietary intakes of children and their parents: a cross-sectional, secondary analysis of families participating in the Family Diet Quality Study. J Hum Nutr Diet. (2015) 28:443–51. 10.1111/jhn.12261 25130863

[31] Wardle J Carnell S Cooke L. Parental control over feeding and children's fruit and vegetable intake: How are they related? J Am Diet Assoc. (2005) 105:227–32. 10.1016/j.jada.2004.11.006 15668680

[32] Fisher JO Mitchell DC Smiciklas-Wright H Birch LL. Parental influences on young girls' fruit and vegetable, micronutrient, and fat intakes. J Am Diet Assoc. (2002) 102:58–64. 10.1016/S0002-8223(02)90017-9 11794503 PMC2530939

[33] Elfhag K Tholin S Rasmussen F. Consumption of fruit, vegetables, sweets and soft drinks are associated with psychological dimensions of eating behaviour in parents and their 12-year-old children. Public Health Nutr. (2008) 11:914–23. 10.1017/S1368980008002371 18498675

[34] Wroten KC O'Neil CE Stuff JE Liu Y Nicklas TA. Resemblance of dietary intakes of snacks, sweets, fruit, and vegetables among mother-child dyads from low income families. Appetite (2012) 59:316–23. 10.1016/j.appet.2012.05.014 22634195

[35] Hall L Collins CE Morgan PJ Burrows TL Lubans DR Callister R. Children's intake of fruit and selected energy-dense nutrient-poor foods is associated with fathers' intake. J Am Diet Assoc. (2011) 111:1039–44. 10.1016/j.jada.2011.04.008 21703382

[36] Zuercher J Wagstaff D Kranz S. Associations of food group and nutrient intake, diet quality, and meal sizes between adults and children in the same household: a crosssectional analysis of U.S. households. Nutr J (2011) 10:131. 10.1186/1475-2891-10-131 22123043 PMC3281797

[37] Thorsdottir I Gunnarsdottir I Ingolfsdottir SE Palsson G Fruit and vegetable intake. Vitamin C and β-carotene intake and serum concentrations in six-year-old children and their parents. Scand J Food Nutr. (2006) 50:71–6. 10.1080/17482970600774702

[38] Vauthier J-M Lluch A Lecomte E Artur Y Herberth B. Family resemblance in energy and macronutrient intakes: the stanislas family study. Int J Epidemiol. (1996) 25:1030–7. 8921491 10.1093/ije/25.5.1030

[39] Oliveria SA Ellison RC Moore LL Gillman MW Garrahie EJ Singer MR. Parent-child relationships in nutrient intake: the Framingham Children's Study. Am J Clin Nutr. (1992) 56:593–8. 1503074 10.1093/ajcn/56.3.593

[40] Feunekes GIJ de Graaf C Meyboom S van Staveren WA. Food choice and fat intake of adolescents and adults: associations of intakes within social Networks (1998) 27, 645–656. 9808794 10.1006/pmed.1998.0341

[41] Simonen RL Perusse L Rankinen T Rice T Rao DC Bouchard C. Familial aggregation of physical activity levels in the Quebec Family Study. Med Sci Sports Exerc. (2002) 34:1137–42. 10.1097/00005768-200207000-00014 12131254

[42] Fogelholm M Nuutinen O Pasanen M Myöhänen E Säätelä T. Parent-child relationship of physical activity patterns and obesity. Int. J. Obes. (1999) 23:1262–8. 10643682 10.1038/sj.ijo.0801061

[43] Jago R Fox K Page A Brockman R Thompson J. Parent and child physical activity and sedentary time: do active parents foster active children? BMC Public Health (2010) 10:194. 10.1186/1471-2458-10-194 20398306 PMC2868817

[44] Maia J Gomes TN Trégouët D-A Katzmarzyk PT. Familial resemblance of physical activity levels in the Portuguese population. J Sci Med Sport. (2014) 17:381–6. 10.1016/j.jsams.2013.09.004 24140161

[45] Craig CL Cameron C Tudor-Locke C. Relationship between parent and child pedometer-determined physical activity: a sub-study of the CANPLAY surveillance study. Int J Behav Nutr Phys Act. (2013) 10:8. 10.1186/1479-5868-10-8 23331386 PMC3663819

[46] Jacobi D Caille A Borys J-M Lommez A Couet C Charles M-A. Parent-offspring correlations in pedometer-assessed physical activity. PLoS ONE (2011) 6:e29195. 10.1371/journal.pone.0029195 22216207 PMC3247254

[47] Schoeppe S Vandelanotte C Bere E Lien N Verloigne M Kovács É. The influence of parental modelling on children's physical activity and screen time. Does it differ by gender? Eur J Public Health (2017) 27:152–7. 10.1093/eurpub/ckw182 28177458

[48] Fuemmeler BF Anderson CB Mâsse LC. Parent-child relationship of directly measured physical activity. Int J Behav Nutr Phys Act. (2011) 8:17. 10.1186/1479-5868-8-17 21385455 PMC3062578

[49] Seabra AF Mendonça DM Göring HHH Thomis MA Maia JA. Genetic and environmental factors in familial clustering in physical activity. Eur J Epidemiol. (2008) 23:205–11. 10.1007/s10654-008-9222-x 18214693

[50] Gomes T dos Santos FK Garganta RM Kenny DA Katzmarzyk PT Maia JAR. Multi-level modelling of physical activity in nuclear families. Ann Hum Biol. (2014) 41:136–42. 10.3109/03014460.2013.836243 24111979

[51] Yao C Rhodes R. Parental correlates in child and adolescent physical activity: a meta-analysis. Int J Behav Nutr Phys Act. (2015) 12:10. 10.1186/s12966-015-0163-y 25890040 PMC4363182

[52] Davison KK Birch LL. Obesigenic families: parents' physical activity and dietary intake patterns predict girls' risk of overweight. Int J Obes Relat Metab Disord. (2002) 26:1186–93. 10.1038/sj.ijo.0802071 12187395 PMC2530921

[53] Davison KK Francis LA Birch LL Reexamining obesigenic families: parents' obesity-related behaviors predict girls' change in BMI. Obes Res. (2005) 13:1980–90. 10.1038/oby.2005.243 16339130 PMC2530936

[54] Baranowski T Families and health actions. In: Gochman DS, editor. Handbook of Health Behavior Research 1: Personal and Social Determinants (New York, NY: Plenum Press) (1997) p. 179–206.

[55] LeBlanc AG Broyles ST Chaput J-P Leduc G Boyer C Borghese MM. Correlates of objectively measured sedentary time and self-reported screen time in Canadian children. Int J Behav Nutr Phys Act. (2015) 12:38. 10.1186/s12966-015-0197-1 25889903 PMC4381481

[56] Tremblay MS LeBlanc AG Kho ME Saunders TJ Larouche R Colley RC. Systematic review of sedentary behaviour and health indicators in school-aged children and youth. Int J Behav Nutr Phys Act. (2011) 8:98. 10.1186/1479-5868-8-98 21936895 PMC3186735

[57] de Rezende LF Rodrigues Lopes M Rey-López JP Matsudo VK Luiz Odo C. Sedentary behavior and health outcomes: an overview of systematic reviews. PLoS ONE (2014) 9:e105620. 10.1371/journal.pone.0105620 25144686 PMC4140795

[58] Prochaska JJ Sallis JF Long B. A physical activity screening measure for use with adolescents in primary care. Arch Pediatr Adolesc Med. (2001) 155:554–9. 10.1001/archpedi.155.5.554 11343497

[59] World Health Organization Global Recommendations on Physical Activity for Health. World Health Organization (2010). Available online at: http://www.who.int/dietphysicalactivity/factsheet_recommendations/en/26180873

[60] Winkler G Döring A. Validation of a short qualitative food frequency list used in several German large scale surveys. Z Ernahrungswiss. (1998) 37:234–41. 9800314 10.1007/pl00007377

[61] Deutsche Gesellschaft für Ernährung e. V Vollwertig Essen und Trinken Nach den 10 Regeln der DGE (2013). Available onlline at: http://www.dge.de/modules.php?name=Content&pa=showpage&pid=15 (Accessed March 14, 2014).

[62] Niermann C Krapf F Renner B Reiner M Woll A. Family health climate scale (FHC-scale): development and validation. Int J Behav Nutr Phys Act. (2014) 11:30. 10.1186/1479-5868-11-30 24593840 PMC4015295

[63] Niermann CYN Kremers SPJ Renner B Woll A. Family health climate and adolescents' physical activity and healthy eating. a cross-sectional study with mother-father-adolescent triads. PLoS ONE (2015) 10: e0143599. 10.1371/journal.pone.0143599 26606157 PMC4659539

[64] Ekvall G (1996). Organizational climate for creativity and innovation. Eur J Work Organ Psychol. 5:105–123.

[65] Little RJA A test of missing completely at random for multivariate data with missing values. J Am Stat Assoc. (1988) 83:1198–202.

[66] Curran PJ West SG Finch JF The robustness of test statistics to nonnormality and specification error in confirmatory factor analysis. Psychol Methods (1996) 1:16–29.

[67] Hofstetter H Dusseldorp E van Empelen P Paulussen TW. A primer on the use of cluster analysis or factor analysis to assess co-occurrence of risk behaviors. Prevent Med. (2014) 67C:141–6. 10.1016/j.ypmed.2014.07.007 25036437

[68] Punj G Stewart DW Cluster analysis in marketing research: review and suggestions for application. J Market Res. (1983) 134–48.

[69] Ketchen DJ Shook CL The application of cluster analysis in strategic management research: an analysis and critique. Strat Manag J. (1996) 17:441–58.

[70] Backhaus K Erichson B Plinke W Weiber R Multivariate Analysemethoden: Eine anwendungsorientierte Einführung. Berlin; Heidelberg: Springer-Verlag (2013).

[71] Ottevaere C Huybrechts I Benser J De Bourdeaudhuij I Cuenca-Garcia M Dallongeville J. Clustering patterns of physical activity, sedentary and dietary behavior among European adolescents. The HELENA study. BMC Public Health (2011) 11:328. 10.1186/1471-2458-11-328 21586158 PMC3112135

[72] Falkstedt D Möller J Zeebari Z Engström K. Prevalence, co-occurrence, and clustering of health-risk behaviors among people with different socio-economic trajectories. A population-based study. Prev Med. (2016) 93:64–9. 10.1016/j.ypmed.2016.09.017 27663427

[73] de Vries H van't Riet J Spigt M Metsemakers J van den Akker M Vermunt JK. Clusters of lifestyle behaviors. Results from the Dutch SMILE study. Prev Med. (2008) 46:203–8. 10.1016/j.ypmed.2007.08.005 17904212

[74] Graham H Hutchinson J Law C Platt L Wardle H. Multiple health behaviours among mothers and partners in England. Clustering, social patterning and intra-couple concordance. SSM Popul Health (2016) 2:824–33. 10.1016/j.ssmph.2016.10.011 28018962 PMC5165044

[75] Adler NE Boyce T Chesney MA Cohen S Folkman S Kahn RL. Socioeconomic status and health: the challenge of the gradient. Am Psychol. (1994) 49:15–24. 8122813 10.1037//0003-066x.49.1.15

[76] Susan Kahn RL Syme SL Socioeconomic status and health. The challenge of the gradient. Am Psychol. (1994) 49:15–24. 8122813 10.1037//0003-066x.49.1.15

[77] Hanson MD Chen E. Socioeconomic status and health behaviors in adolescence. A review of the literature. J Behav Med. (2007) 30:263–85. 10.1007/s10865-007-9098-3 17514418

[78] Pampel FC Krueger PM Denney JT. Socioeconomic disparities in health behaviors. Annu Rev Sociol. (2010) 36:349–70. 10.1146/annurev.soc.012809.102529 21909182 PMC3169799

[79] Fielding-Singh P. Dining with dad. Fathers' influences on family food practices. Appetite (2017) 117:98–108. 10.1016/j.appet.2017.06.013 28629930

[80] Bianchi SM. Maternal employment and time with children. Dramatic change or surprising continuity? Demography (2000) 37:401–14. 10.1353/dem.2000.0001 11086567

[81] Yeung WJ Sandberg JF Davis-Kean PE Hofferth SL (2001). Children's time with fathers in intact families. J Marriage Family 63, 136–54. 10.1111/j.1741-3737.2001.00136.x

[82] Vereecken CA Keukelier E Maes L. Influence of mother's educational level on food parenting practices and food habits of young children. Appetite (2004) 43:93–103. 10.1016/j.appet.2004.04.002 15262022

[83] Davison KK Lawson HA Coatsworth JD. The Family-Centered Action Model of Intervention Layout and Implementation (FAMILI):the example of childhood obesity. Health Promot Pract. (2012) 13:454–61. 10.1177/1524839910377966 21632465

[84] Sung-Chan P Sung YW Zhao X Brownson RC. Family-based models for childhood-obesity intervention. A systematic review of randomized controlled trials. Obes Rev. (2013) 14:265–78. 10.1111/obr.12000 23136914

[85] Kitzmann KM Dalton WT Buscemi J Beyond parenting practices. family context and the treatment of pediatric obesity^*^ Fam Relat. (2008) 57:13–23. 10.1111/j.1741-3729.2007.00479.x

[86] Sleddens EFC Gubbels JS Kremers SPJ van der Plas E Thijs C. Bidirectional associations between activity-related parenting practices, and child physical activity, sedentary screen-based behavior and body mass index: a longitudinal analysis. Int J Behav Nutr Phys Act. (2017) 14:89. 10.1186/s12966-017-0544-5 28683749 PMC5501263

[87] Cox MJ Paley B Understanding families as systems. Curr Dir Psychol Sci. (2003) 12:193–6. 10.1111/1467-8721.01259

[88] Berge JM Rowley S Trofholz A Hanson C Rueter M MacLehose RF. Childhood obesity and interpersonal dynamics during family meals. Pediatrics (2014) 134:923–32. 10.1542/peds.2014-1936 25311603 PMC4210801

[89] Berge JM Wall M Larson N Loth KA Neumark-Sztainer D. Family functioning. Associations with weight status, eating behaviors, and physical activity in adolescents J Adolesc Health. (2013) 52:351–7. 10.1016/j.jadohealth.2012.07.006 23299010 PMC3580029

[90] Haines J Rifas-Shiman SL Horton NJ Kleinman K Bauer KW Davison KK. Family functioning and quality of parent-adolescent relationship. Cross-sectional associations with adolescent weight-related behaviors and weight status. Int J Behav Nutr Phys Act. 13:68. 10.1186/s12966-016-0393-7 27301414 PMC4908682

[91] Prochaska JJ Spring B Nigg CR. Multiple health behavior change research: an introduction and overview. Prev Med. (2008) 46:181–8. 10.1016/j.ypmed.2008.02.001 18319098 PMC2288583

[92] Meader N King K Wright K Graham HM Petticrew M Power C. Multiple risk behavior interventions. Meta-analyses of RCTs. Am J Prev Med. (2017) 53:e19–30. 10.1016/j.amepre.2017.01.032 28258777

[93] Prochaska JO. Multiple health behavior research represents the future of preventive medicine. Prev Med. (2008) 46:281–5. 10.1016/j.ypmed.2008.01.015 18319100

